# Incidence and risk factors for insulinoma diagnosed in dogs under primary veterinary care in the UK

**DOI:** 10.1038/s41598-025-86782-6

**Published:** 2025-01-20

**Authors:** Kasper Kraai, Dan G. O’Neill, Lucy J. Davison, Dave C. Brodbelt, Sara Galac, Floryne O. Buishand

**Affiliations:** 1 Department of Clinical Sciences, Faculty of Veterinary Medicine, Utrecht University, Utrecht, 3584 CM The Netherlands; 2 Department of Pathobiology and Population Sciences, Royal Veterinary College, Hatfield, AL9 7TA UK; 3 Department of Clinical Science and Services, Royal Veterinary College, Hatfield, AL9 7TA UK

**Keywords:** Canine, Demographic risk factors, Epidemiology, Pancreas, Pancreatic neuroendocrine neoplasm, VetCompass., Neuroendocrine cancer, Cancer epidemiology

## Abstract

Insulinoma is the most common pancreatic tumor diagnosed in dogs. This study aimed to report incidence risk, breed predispositions and other demographic risk factors for insulinoma diagnosed in dogs under primary veterinary care in the UK. The VetCompass Program supports research on anonymized electronic health records (EHRs) from dogs under UK veterinary care. This study included all VetCompass EHRs from dogs under primary veterinary care during 2019. Multivariable logistic regression analysis was used to evaluate demographic risk factors for insulinoma diagnosis. Of 2,250,741 study dogs, 278 were confirmed as insulinoma cases at any date. The estimated 2019 incidence risk was 0.003% (95% CI 0.002–0.004%). Compared to crossbreeds, predisposed breeds included Dogue de Bordeaux, German Pointer, Flat Coated Retriever, Boxer and West Highland White Terrier. The Labrador Retriever showed decreased odds for insulinoma diagnosis. Additionally, being a terrier breed and being a breed predisposed to other endocrine cancers were associated with increased odds for insulinoma diagnosis. Other risk factors associated with increased odds for insulinoma diagnosis included being female neutered, being 9 - <15 years of age, having an adult median bodyweight of 20 - <30 kg and having a bodyweight above the median for the sex/breed. This is the first study to report the epidemiology of canine insulinoma in dogs under primary veterinary care, resulting in crucial leads for further research in the epidemiology and etiology of canine insulinoma and possible links of canine insulinoma with other canine endocrine cancers. Additionally, the results can aid veterinarians to identify dogs at greater risk of insulinoma.

**Supplementary Information:**

The online version contains supplementary material available at 10.1038/s41598-025-86782-6.

## Introduction

Insulinoma is the most common neuroendocrine tumor of the pancreas in dogs and humans^1,2^. In both species, neoplastic insulinoma β-cells secrete insulin in an uncontrolled fashion, leading to hyperinsulinemia-induced hypoglycemia. Human malignant insulinoma and spontaneous canine insulinoma share similar histological features, molecular characteristics and responses to multimodal treatment protocols^3–7^. Hence, canine insulinoma has been validated as a research model for human malignant insulinoma^7^. The advantages of using a canine insulinoma model over a rodent model include the fact that dogs more closely represent the genetics of humans than rodents and develop insulinomas spontaneously in contrast to genetically induced insulinomas in rodents. Additionally, dogs share their living environment with humans and are exposed to similar environmental factors. Moreover, spontaneous insulinomas in dogs provide a model with intact host immunity, as well as natural tumor heterogeneity and microenvironment. However, further research into the genetic background of these tumors is needed to determine whether they share the same drivers as those found in human patients^8^.

In humans, the reported incidence of insulinoma ranges from 1 to 4 cases per million population per year^9^. The incidence risk for canine insulinoma has not been formally reported but has been suggested to be higher than for human insulinoma^6^. Despite this, canine insulinoma is considered an under-recognized disease in primary care veterinary practice because clinical hypoglycemia can be intermittent and non-specific, and can mimic clinical signs of other endocrine, neurological and/or cardiovascular diseases^10,11^.

Although most insulinoma in humans occur sporadically, 5–10% of human cases are linked with Multiple Endocrine Neoplasia type 1 (MEN1), a genetic syndrome characterized by simultaneous occurrence of neoplasms in multiple endocrine organs. MEN1 is characterized by the simultaneous occurrence of at least two endocrine tumors arising from either the pancreas, parathyroid, and/or pituitary glands^12^. In veterinary medicine, MEN is preferably called Concurrent Endocrine Neoplasia (CEN), although reported instances of this syndrome in dogs are rare^13^.

In humans up to 30% of pancreatic neuroendocrine neoplasms (panNENs) are functional and the clinical syndrome is related to the specific hormone overproduction, with the remainder of panNENs being non-functional^14^. Several risk factors are reported as associated with the occurrence of human sporadic panNENs. Personal history of diabetes mellitus and a family history of cancer, and heavy smoking and heavy alcohol consumption have been linked with increased risk of pancreatic neuroendocrine tumors in various single institutional case-control studies^15–18^. In a multinational European case-control study, only non-recent onset diabetes mellitus was associated with increased occurrence of pancreatic neuroendocrine tumors^19^. However, a major limitation of these studies is that they focused on risk factors for pancreatic neuroendocrine tumors in general, with only small subgroups of insulinoma patients included within those overall study populations, precluding conclusions regarding specific risk factors for insulinoma. Only one study performed in a Chinese population looked specifically at insulinoma, and reported a family history of pancreatic endocrine tumors or other cancers and living in a rural area as risk factors for insulinoma^20^.

The literature on insulinoma in dogs is dominated by case reports and case series that focused on clinical findings and outcomes, with very little published work that included large numbers of dogs to explore breed predisposition and other risk factors. Most cases of insulinoma in these case series were diagnosed in medium and large breed dogs, although some cases were also described in small breed dogs. Apparently overrepresented breeds in these studies included Boxer, terrier and retriever breeds, although a clear breed predisposition for insulinoma has not been established^10,21–25^. Similarly, no sex predisposition has been reported and no data are available on other risk factors for the diagnosis of insulinoma in dogs^22^. The reported median age at insulinoma diagnosis ranged from nine to eleven years^10,11,21–25^.

‘Big Data’ resources and data-sharing initiatives have proven hugely valuable in advancing the understanding of epidemiology of uncommon and rare diseases^26,27^. The VetCompass Program, a UK nationwide veterinary surveillance system, shares anonymized veterinary electronic health records (EHRs) from over 14 million dogs and has been validated as a valuable research resource by over 140 peer reviewed publications to date^28^. Using VetCompass EHRs, the current study aimed to report the annual (2019) prevalence and incidence risk as well as demographic risk factors for insulinoma diagnosis in dogs under primary veterinary care in the UK. The study placed particular focus on associations with breed. These results could assist veterinary practitioners by providing a stronger evidence base to improve diagnosis and management of insulinoma in dogs.

## Results

### Demography

**Fig. 1:**
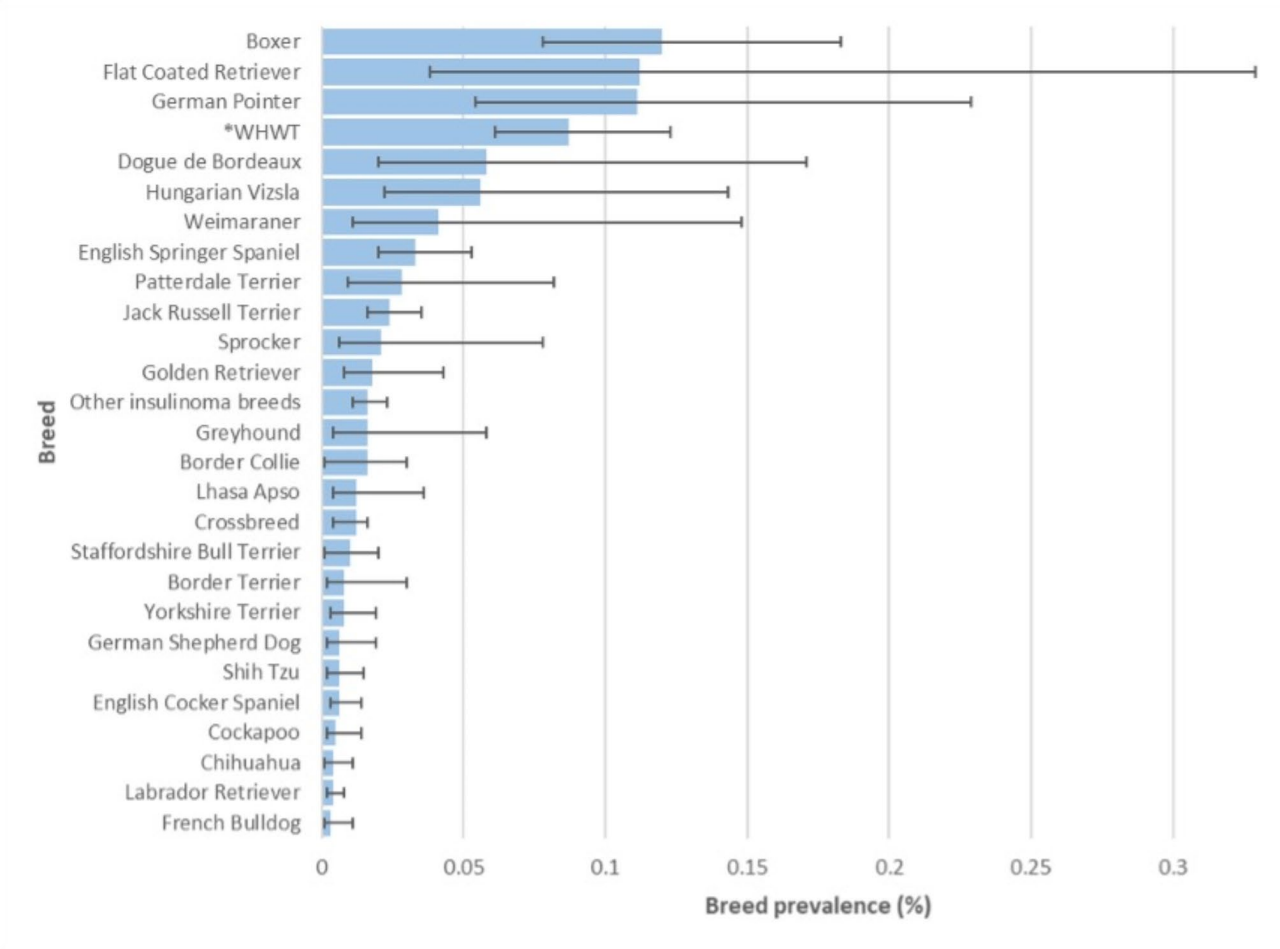
Available-lifetime breed prevalence of insulinoma diagnosis in dogs under primary veterinary care at practices in the VetCompass Program in the UK in 2019. The error bars show the 95% confidence interval. Only dog breeds with ≥ 2 insulinoma cases are displayed. *WHWT West Highland White Terrier.

From the study population of 2,250,741 dogs under primary veterinary care in 2019, 1,881 (0.08%) candidate insulinoma cases were identified. Manual review of all candidate cases confirmed 278 (14.8%) as insulinoma cases that met the inclusion criteria at any date in the available EHRs up to August 31, 2023, resulting in an available-lifetime prevalence for insulinoma diagnosis of 0.012% (95% CI 0.011–0.014). The initial insulinoma diagnoses of the 278 cases were made between 2015 and 2023 and within the 278 cases, 110 cases were diagnosed at any date up to 31 December 2019, resulting in an annual (2019) prevalence of 0.004% (95% CI 0.003–0.005). Sixty-three cases were first diagnosed in 2019, giving an annual (2019) incidence risk for insulinoma diagnosis of 0.003% (95% CI 0.002–0.004). Of breeds with ≥ 2 insulinoma cases, the breeds with the highest available-lifetime prevalence were Boxer (*n* = 21, 0.12%, 95% CI 0.08–0.18%), Flat Coated Retriever (*n* = 3, 0.11%, 95% CI 0.04–0.33%), German Pointer (*n* = 7, 0.11%, 95% CI 0.05–0.23%) and West Highland White Terrier (*n* = 31, 0.09%, 95% CI 0.06–0.12%) (Fig. 1).

Of the 278 insulinoma cases diagnosed up until August 31, 2023, 123 (44.24%) were male (with 52.03% neutered), 153 (55.04%) were female (with 73.20% neutered), and the sex was not recorded in 2 cases (0.72%). Age at the time of diagnosis was available for 268/278 (96.40%) dogs. The median age at first diagnosis was 10.4 years (interquartile range [IQR] 8.7–12.2, range 3.2–16.2). Median adult bodyweight was available for 252/278 (90.65%) dogs. The median of the median adult bodyweight was 18.9 kg (IQR 9.3–28.4, range 3.4–68.3). The most commonly diagnosed breeds with insulinoma were crossbreed, West Highland White Terrier, and Jack Russell Terrier (Table 1).

**Table 1:** Descriptive and univariable logistic regression results for dog breeds with ≥ 2 cases of insulinoma as a risk factor for insulinoma diagnosis during 2019 in dogs under primary veterinary care in the VetCompass Program in the UK. Column percentages shown in brackets.

Breed	Insulinoma cases, *n* (%)	Non-insulinoma cases, *n* (%)	Odds ratio	95% CI*	Category *P*-value	Variable *P*-value
Crossbreed	67 (24.10)	178,996 (23.87)	Ref**			**< 0.001**
Boxer	21 (7.55)	5,917 (0.79)	9.51	5.82–15.54	**< 0.001**	
Flat Coated Retriever	3 (1.08)	889 (0.12)	9.04	2.84–28.80	**< 0.001**	
German Pointer	7 (2.52)	2,030 (0.27)	9.21	4.22–20.08	**< 0.001**	
West Highland White Terrier	31 (11.15)	11,968 (1.60)	6.94	4.53–10.62	**< 0.001**	
Dogue de Bordeaux	3 (1.08)	1,687 (0.22)	4.75	1.49–15.11	**0.008**	
Hungarian Vizsla	4 (1.44)	2,379 (0.32)	4.50	1.64–12.35	**0.004**	
Weimaraner	2 (0.72)	1,635 (0.22)	3.27	0.80-13.36	0.099	
English Springer Spaniel	17 (6.12)	17,084 (2.28)	2.66	1.56–4.53	**< 0.001**	
Patterdale Terrier	3 (1.08)	3,518 (0.47)	2.28	0.72–7.25	0.163	
Jack Russell Terrier	24 (8.63)	33,602 (4.48)	1.91	1.20–3.04	**0.007**	
Sprocker	2 (0.72)	3,160 (0.42)	1.69	0.41–6.91	0.464	
Golden Retriever	5 (1.80)	9,253 (1.23)	1.44	0.58–3.58	0.428	
Border Collie	10 (3.60)	20,430 (2.72)	1.31	0.67–2.54	0.429	
Greyhound	2 (0.72)	4,200 (0.56)	1.27	0.31–5.19	0.738	
Lhasa Apso	3 (1.08)	8,348 (1.11)	0.96	0.30–3.05	0.945	
Staffordshire Bull Terrier	9 (3.24)	31,394 (4.19)	0.77	0.38–1.54	0.452	
Border Terrier	2 (0.72)	8,216 (1.10)	0.65	0.16–2.65	0.548	
Yorkshire Terrier	4 (1.44)	17,741 (2.37)	0.60	0.22–1.65	0.325	
German Shepherd Dog	3 (1.08)	15,680 (2.09)	0.51	0.16–1.63	0.255	
English Cocker Spaniel	6 (2.16)	32,325 (4.31)	0.50	0.22–1.14	0.100	
Shih Tzu	4 (1.44)	22,426 (2.99)	0.48	0.17–1.31	0.150	
Cockapoo	4 (1.44)	24,466 (3.26)	0.44	0.16–1.20	0.107	
Labrador Retriever	6 (2.16)	51,572 (6.88)	0.31	0.14–0.72	**0.006**	
Chihuahua	3 (1.08)	27,005 (3.60)	0.30	0.09–0.94	**0.040**	
French Bulldog	2 (0.72)	22,268 (2.97)	0.24	0.06–0.98	**0.047**	
Other breeds	31 (11.15)	191,811 (25.57)	0.43	0.28–0.66	**< 0.001**	
Total	278 (100.02)	750,000 (100.01)				

*CI, confidence interval.

** Ref, reference category indicates the baseline category to which the other categories were compared.

Of the random sample of 750,000 non-insulinoma cases, 388,113 (51.75%) were male (with 42.80% neutered), 355,532 (47.40%) were female (with 44.80% neutered) and the sex was not recorded in 6,355 cases (0.85%). The median age at 31 December 2019 was available for 744,152/750,000 (99.22%). The median age at 31 December 2019 was 5.2 years (IQR 2.3-9.0, range 0.0-24.8). The median adult bodyweight was available for 513,735/750,000 (68.50%) dogs. The median of the median adult bodyweight was 13.7 kg (IQR 8.4–24.4, range 0.4–95.0). The most common breeds among non-insulinoma cases were crossbreed, Labrador Retriever, and Jack Russell Terrier (Table 1).

## Risk factor analysis

Univariable binary logistic regression modelling identified 14 variables liberally associated with insulinoma diagnosis at any date in the available records up to August 31, 2023 (*P* < 0.20): sex/neuter, age, median adult bodyweight, purebred status, breed, median adult bodyweight in relation to the median for the sex/breed, terrier breed, breed predisposed for mammary gland cancer, breed predisposed for parathyroid cancer, breed predisposed for thyroid cancer, breed predisposed for parathyroid and/or thyroid cancer, breed predisposed for parathyroid and/or thyroid and/or pituitary/adrenal cancer, breed predisposed for reproductive organ cancer and breed predisposed for testicular and/or ovarian cancer (Tables 1 and 2).

**Table 2:** Descriptive and univariable logistic regression results for risk factors evaluated for 278 dogs with the diagnosis insulinoma compared to randomly selected controls (*n* = 750,000) attending UK VetCompass primary care veterinary practices in 2019. Column percentages shown in brackets.

Variable	Insulinoma Case No. (%)	Non-case No. (%)	Odds ratio	95% CI*	Category *P*-value	Variable *P*-value
Sex/neuter						**< 0.001**
Female entire	41 (14.75)	196,469 (26.20)	Ref**			
Female neutered	112 (40.29)	159,063 (21.21)	3.37	2.36–4.83	**< 0.001**	
Male entire	59 (21.22)	221,981 (29.60)	1.27	0.86–1.90	0.234	
Male neutered	64 (23.02)	166,132 (22.15)	1.85	1.25–2.73	**0.002**	
Unrecorded	2 (0.72)	6,355 (0.85)	1.51	0.37–6.24	0.571	
Age at time of diagnosis (insulinoma cases) and age at December 31, 2019 (non-cases insulinoma)						**< 0.001**
0 - <6	14 (5.04)	413,785 (55,18)	0.08	0.05–0.14	**< 0.001**	
6 - <9	61 (21.94)	144,923 (19.32)	Ref			
9 - <12	121 (43.53)	108,324 (14.44)	2.65	1.95–3.61	**< 0.001**	
12 - <15	68 (24.46)	61,082 (8.14)	2.65	1.87–3.74	**< 0.001**	
≥ 15	4 (1.44)	16,049 (2.14)	0.59	0.22–1.63	0.310	
Unrecorded	10 (3.60)	5,837 (0.78)	4.07	2.08–7.95	**< 0.001**	
Median adult bodyweight						**< 0.001**
< 10 kg	74 (26.62)	180,547 (24.07)	Ref			
10 - <20 kg	57 (20.50)	158,608 (21.15)	0.88	0.62–1.24		
20 - <30 kg	72 (25.90)	99,861 (13.31)	1.76	1.27–2.43	**< 0.001**	
≥ 30 kg	49 (17.63)	74,719 (9.96)	1.60	1.12–2.30	**0.011**	
Unrecorded	26 (9.35)	236,265 (31.50)	0.27	0.17–0.42	**< 0.001**	
Purebred						0.158
Crossbreed	67 (24.10)	178,929 (23.86)	Ref			
Designer	9 (3.24)	50,018 (6.67)	0.48	0.24–0.96	**0.039**	
Purebred	202 (72.66)	516,602 (68.88)	1.04	0.79–1.38	0.759	
Unrecorded	0 (0.0)	4,451 (0.59)	0	0		
Median adult bodyweight in relation to the median for the sex/breed						**< 0.001**
At or below median adult bodyweight for the sex/breed	93 (33.45)	258,309 (34.44)	Ref			
Above median adult bodyweight for the sex/breed	159 (57.19)	254,911 (33.99)	1.73	1.34–2.24	**< 0.001**	
Unrecorded	26 (9.35)	236,780 (31.57)	0.31	0.20–0.47	**< 0.001**	
Terrier						**< 0.001**
Non-terrier	200 (71.94)	628,337 (83.78)	Ref			
Terrier	78 (28.06)	121,663 (16.22)	2.01	1.55–2.62	**< 0.001**	
Breed predisposed for mammary gland cancer						0.059
Not predisposed	174 (62.59)	509,167 (67.89)	Ref			
Predisposed	104 (37.41)	240,833 (32.11)	1.26	0.99–1.61	0.059	
Breed predisposed for parathyroid cancer						**< 0.001**
Not predisposed	226 (81.29)	676,957 (90.26)	Ref			
Predisposed	52 (18.71)	73,043 (9.74)	2.95	2.01–4.33	**< 0.001**	
Breed predisposed for thyroid cancer						**< 0.001**
Not predisposed	249 (89.57)	721,508 (96.20)	Ref			
Predisposed	29 (10.43)	28,492 (3.80)	2.13	1.58–2.88	**< 0.001**	
Breed predisposed for pituitary/adrenal cancer						0.281
Not predisposed	208 (74.82)	581,431 (77.52)	Ref			
Predisposed	70 (25.18)	168,569 (22.48)	1.16	0.89–1.52	0.281	
Breed predisposed for parathyroid and/or thyroid cancer						**< 0.001**
Not predisposed	224 (80.58)	669,361 (89.25)	Ref			
Predisposed	54 (19.42)	80,639 (10.75)	2.00	1.49–2.69	**< 0.001**	
Breed predisposed for parathyroid and/or thyroid and/or pituitary/adrenal cancer						**0.022**
Not predisposed	182 (65.47)	537,455 (71.66)	Ref			
Predisposed	96 (34.53)	212,545 (28.34)	1.33	1.04–1.71	**0.022**	
Breed predisposed for reproductive endocrine organ cancer***						0.073
Not predisposed	167 (60.07)	488,980 (65.20)	Ref			
Predisposed	111 (39.93)	261,020 (34.80)	1.25	0.98–1.58	0.073	
Breed predisposed for testicular and/or ovarian cancer						0.084
Not predisposed	241 (86.69)	673,744 (89.83)	Ref			
Predisposed	37 (13.31)	76,256 (10.17)	1.36	0.96–1.92	0.084	
Breed predisposed for one or multiple types of endocrine cancer						0.857
Not predisposed	157 (56.47)	419,527 (55.94)	Ref			
Predisposed	121 (43.53)	330,473 (44.06)	0.98	0.77–1.24	0.857	

*CI, confidence interval.

** Ref, reference category indicates the baseline category to which the other categories were compared.

***Reproductive endocrine organs include mammary glands, testicles and ovaria.

The final breed-focused multivariable logistic regression model retained four variables: breed, sex/neuter, age and median adult bodyweight in relation to the median for the sex/breed (Table 3). The area under the Receiver Operating Characteristic (ROC) curve of the final breed-focused model was 0.868, indicating good discrimination. Seven breeds had increased odds for insulinoma diagnosis compared to crossbreed. The Dogue de Bordeaux, German Pointer, Flat Coated Retriever and Boxer showed the highest predisposition. The Labrador Retriever showed decreased odds for insulinoma diagnosis compared to the crossbreed. Increased odds for insulinoma diagnosis was identified for female neutered dogs compared with female entire dogs, dogs with a median adult bodyweight above the median for the sex/breed compared to dogs with a median adult bodyweight at or below the median for the sex/breed, and dogs aged between 9 - <12 years and 12 - <15 years compared to dogs aged between 6 - <9 years. Dogs aged between 0 - <6 years compared to dogs aged between 6 - <9 years showed decreased odds.

**Table 3:** Results of final breed-focused multivariable logistic regression model for the risk factors associated with insulinoma diagnosis in dogs under primary veterinary care in the VetCompass Program in the UK.

Variable	Odds ratio	95% CI*	Category *P*-value	Variable *P*-value
Breed				**< 0.001**
Crossbreed	Ref**			
Dogue de Bordeaux	9.33	2.90-29.98	**< 0.001**	
German Pointer	8.73	4.00-19.09	**< 0.001**	
Flat Coated Retriever	8.24	2.58–26.33	**< 0.001**	
Boxer	8.22	5.02–13.47	**< 0.001**	
Hungarian Vizsla	6.29	2.29–17.31	**< 0.001**	
West Highland White terrier	4.22	2.74–6.48	**< 0.001**	
Weimaraner	2.58	0.63–10.57	0.187	
English Springer Spaniel	2.05	1.20–3.49	**0.009**	
Patterdale terrier	2.00	0.63–6.36	0.241	
Cockapoo	1.35	0.49–3.74	0.563	
Jack Russell terrier	1.33	0.83–2.12	0.236	
French Bulldog	1.02	0.25–4.23	0.982	
Shih Tzu	0.49	0.18–1.36	0.171	
Chihuahua	0.46	0.14–1.46	0.185	
Labrador Retriever	0.26	0.11–0.60	**0.002**	
Other breeds	0.60	0.43–0.83	**0.002**	
Sex/neuter				**0.019**
Female entire	Ref			
Female neutered	1.50	1.04–2.17	**0.029**	
Male entire	1.21	0.81–1.80	0.354	
Male neutered	0.91	0.61–1.36	0.652	
Unrecorded	1.02	0.24–4.33	0.981	
Mean adult bodyweight in relation to the median for the sex/breed				**< 0.001**
At or below median adult bodyweight for the sex/breed	Ref			
Above median adult bodyweight for the sex/breed	1.44	1.11–1.86	**0.006**	
Unrecorded	0.36	0.21–0.62	**< 0.001**	
Age				**< 0.001**
0 - <6	0.11	0.06–0.20	**< 0.001**	
6 - <9	Ref			
9 - <12	2.55	1.87–3.48	**< 0.001**	
12 - <15	2.51	1.77–3.57	**< 0.001**	
15+	0.60	0.22–1.64	0.316	
Unrecorded	14.89	6.42–34.56	**< 0.001**	

*CI, confidence interval.

** Ref, reference category indicates the baseline category to which the other categories were compared.

As described in the methods, breed-derived variables were introduced individually to replace *breed* in the final breed-focused model. Five other variables were identified as significantly associated with insulinoma diagnosis: median adult bodyweight (20 - <30 kg had increased odds compared to < 10 kg), terrier breed, breed predisposed for parathyroid cancer, breed predisposed for thyroid cancer and breed predisposed for parathyroid and/or thyroid cancer (Table 4).

**Table 4:** Results for variables derived from breed or correlated with breed that were used to replace breed in the final breed-focused multivariable logistic regression model for risk factors associated with insulinoma diagnosis in dogs under primary veterinary care in the VetCompass Program in the UK.

Variable	Odds ratio	95% CI*	Category *P*-value	Variable *P*-value
Median adult bodyweight				**< 0.001**
< 10 kg	Ref**			
10–20 kg	0.89	0.63–1.26	0.505	
20–30 kg	1.50	1.08–2.08	**0.015**	
> 30 kg	1.42	0.99–2.05	0.057	
Unrecorded	0.33	0.19–0.56	**< 0.001**	
Purebred				0.999
Crossbreed	Ref			
Designer	0.96	0.48–1.2	0.899	
Not applicable	0	0		
Purebred	1.01	0.77–1.34	0.936	
Terrier				**0.024**
Non-terrier	Ref			
Terrier	1.36	1.05–1.77	**0.022**	
Breed predisposed for mammary gland cancer				0.670
Not predisposed	Ref			
Predisposed	0.95	0.75–1.22	0.695	
Breed predisposed for parathyroid cancer				**< 0.001**
Not predisposed	Ref			
Predisposed	1.92	1.42–2.60	**< 0.001**	
Breed predisposed for thyroid cancer				**< 0.001**
Not predisposed	Ref			
Predisposed	2.89	1.97–4.25	**< 0.001**	
Breed predisposed for parathyroid and/or thyroid cancer				**< 0.001**
Not predisposed	Ref			
Predisposed	1.83	1.36–2.46	**< 0.001**	
Breed predisposed for parathyroid, thyroid and pituitary/adrenal cancer				0.813
Not predisposed	Ref			
Predisposed	1.03	0.80–1.32	0.813	
Breed predisposed for reproductive endocrine organ cancer***				0.628
Not predisposed	Ref			
Predisposed	0.94	0.74–1.20	0.628	
Breed predisposed for testicular and ovarian cancer				0.073
Not predisposed	Ref			
Predisposed	1.37	0.97–1.94	0.072	

*CI confidence interval.

** Ref, reference category indicates the baseline category to which the other categories were compared.

***Reproductive endocrine organs include mammary glands, testicles and ovaria.

## Discussion

This is the largest study to date to report the frequency and risk factors for diagnosis with insulinoma in dogs under primary veterinary care. Several breed predispositions and other risk factors were identified that can help improve recognition of insulinoma in primary veterinary care and may contribute to translational insulinoma studies that can inform human medicine.

The current study identified an annual prevalence of 0.004% in 2019 for insulinoma in dogs under primary veterinary care in the UK and a corresponding annual incidence risk of newly diagnosed insulinoma of 0.003%. Additionally, we found an available-lifetime prevalence for the diagnosis insulinoma of 0.012%. No previous studies have reported on data from primary veterinary care practices that could be useful for comparison to the current results. Previously, Capodanno et al., 2018 reported that the only academic veterinary referral hospital in the Netherlands recorded 10 new referral cases of canine insulinoma yearly from an overall two million dogs in the Netherlands, but their study was not designed to calculate an annual incidence risk, so direct comparisons between their finding and the current study results cannot be made^6^. Human studies have reported incidence rates of insulinoma ranging from 0.0 to four cases per million population per year (0.0000-0.0004%), which is ten times lower than reported in the current study for dogs^29–31^. Diagnosing canine insulinoma can be challenging because the clinical signs are often non-specific and can mimic other neurological, endocrine and metabolic conditions^10,11^. Furthermore, 68Gallium DOTA-(Tyr3)-octreotate positron emission tomography/computed tomography, a highly sensitive and specific imaging technique frequently used in the diagnostic work-up of insulinomas in humans, is not available in veterinary medicine^32^. Therefore, some or even many true insulinoma cases may have remained unascertained in the current population studied and consequently the current prevalence figures may be an underestimate. Proportional malignancy of human insulinoma is reportedly much lower than for canine insulinoma, with only 5–16% of human insulinoma considered malignant compared to almost all canine insulinoma considered malignant based on their metastatic behavior^5,31^. A lack of understanding of human malignant insulinoma tumorigenesis can be partly attributed to the low annual incidence rate of human malignant insulinoma. The up to ten times higher estimated annual incidence rate of insulinoma in dogs compared to humans combined with the substantially higher rates of malignancy of insulinoma in dogs underpins the value of spontaneous canine insulinoma as a translational study model for human malignant insulinoma because canine insulinoma samples are more readily available for molecular studies, unlike human malignant insulinoma samples.

The current study identified seven breeds with predisposition for insulinoma diagnosis: Dogue de Bordeaux, German Pointer, Flat Coated Retriever, Boxer, Hungarian Vizsla, West Highland White Terrier and English Springer Spaniel. Among the seven breeds identified with increased odds of insulinoma diagnosis, six breeds demonstrated ultra-predispositions, defined as having over four-times higher odds compared to crossbreed dogs^33^. Of these ultra-predisposed breeds, the Dogue the Bordeaux, Flat Coated Retriever and German Pointer have not been previously identified as overrepresented for insulinoma^10,21–25^. The finding of ultra-predispositions for the Boxer and West Highland White Terrier aligns with previous descriptive studies that generally reported these breeds as overrepresented for insulinoma^10,21–25^. Previous insulinoma studies, however, were descriptive and did not statistically compare breed proportional risk to a control or denominator population, making direct comparison to the current work difficult. The Boxer and Flat Coated Retriever are frequently reported with increased odds for cancers in general so the findings of the current study may represent an extension of this overall cancer predilection^34–39^.

The current study also identified an association between being a terrier breed in general and insulinoma diagnosis. Terrier breeds have previously been reported to cluster in one of the 23 breed clades that were identified in a genomic analysis including 161 breeds that investigated the influence of geographic origin, migration and hybridization on modern dog breed development^40^. Knowledge of predisposed breeds may aid in insulinoma recognition and contributes to further understanding of canine insulinoma genetics. Especially for a rarely diagnosed disease like canine insulinoma, future genomic studies into genetic variants that contribute to canine insulinoma development could benefit from comparing between breeds that are predisposed to insulinoma versus breeds that are protected against insulinoma. Although the current study did not aim to identify breeds with protection to insulinoma, the Labrador Retriever was identified with relative protection against insulinoma diagnosis amongst those breeds with 2 or more insulinoma cases diagnosed. This finding would need confirmation in future work using a different data source to avoid Type I error (false positive) but, if confirmed, the Labrador Retriever breed could represent a useful negative control for future genetic studies. There may be other breeds with relative protection, especially breeds with no insulinoma diagnosed cases in this population, but relative protection was not the primary focus of the current study. Future breed-focused research, including even greater numbers of cases, could provide new insights on breeds protected for insulinoma diagnosis.

A strong association was identified in the current study between sex/neuter status and insulinoma diagnosis that has not been reported previously. Altered hormonal patterns could explain insulinoma predisposition in female neutered dogs compared to female entire dogs. Female neutered dogs have reduced serum estrogen levels, whereas cyclical estrogen exposure has been reported to be a protective factor against pancreatic neuroendocrine tumorigenesis, although a definitive role for estrogen exposure cannot be established by our data^41^. Another potential explanation for predisposition of female neutered dogs for insulinoma is that the median age at insulinoma diagnosis was 10 years and the proportion of female dogs that is neutered increases over time. Age was strongly associated with the odds of insulinoma diagnosis. Dogs aged 9 - <15 years had increased odds for insulinoma diagnosis and dogs aged 3 - <6 years had decreased odds for insulinoma diagnosis compared to dogs aged 6 - <9 years. This finding is reasonably consistent with previous studies, that report a median age at time of first diagnosis ranging 9–11 years^10,11,21–25,42^.

Absolute bodyweight was strongly associated with the odds of insulinoma diagnosis. Dogs with a median adult bodyweight of 20–30 kg had increased odds for insulinoma diagnosis compared with dogs with a median adult bodyweight of < 10 kg. This finding is consistent with previous insulinoma studies that report medium and large breed dogs (> 25 kg) as overrepresented^42^.

Within breeds, dogs with a median adult bodyweight above the median for the sex/breed had increased odds for insulinoma diagnosis. Previous studies in both humans and dogs have reported associations between higher body condition score and/or obesity and certain types of cancer, including human pancreatic neuroendocrine tumors^16,20,43,44^. Similarly, the current results could also be interpreted to offer some support for increased risk of insulinoma in overweight dogs. However, caution should be exercised with this interpretation as the current study used the median adult bodyweight compared to the median for the sex/breed and it may be that higher bodyweight reflects larger individual dogs rather than higher body condition score^45^. Additionally, dogs with insulinoma tend to gain weight after the condition develops due to anabolic effects of insulin on metabolism. Therefore, weight gain leading to a higher median weight compared to the median for the sex/breed could be a consequence rather than a cause of insulinoma^42^.

Until now, low case numbers in previous works have limited the evidence of CEN in veterinary medicine; only one dog has been reported in the literature to have concurrent insulinoma and pituitary adenoma, resembling human MEN1 syndrome^12,13^. MEN1 is caused by a germline mutation in the *MEN1* gene and is inherited as an autosomal disorder. Although Beatrice et al. (2018) reported only one dog resembling MEN1 syndrome, they did find other combinations of insulinoma with concurrent endocrine tumors in dogs as well, including insulinoma with pheochromocytoma, or adrenocortical hyperplasia^13^. Additionally, Kiupel et al. (2000) reported an insulinoma, bilateral adrenocortical adenocarcinomas and an aortic paraganglioma in a 12-year-old crossbreed^46^. In contrast to these sporadic earlier CEN reports based on smaller study populations, the current study benefited from data on a large denominator of dogs, which supported identification of associations between being a breed predisposed for certain types of endocrine cancer and insulinoma diagnosis. The current study demonstrated that breeds predisposed for parathyroid-, thyroid- and parathyroid and/or thyroid cancer had increased odds for insulinoma diagnosis. This association should be interpreted with caution because the current study included insulinoma diagnoses only from dogs in the UK, where genetic bottleneck effects and region-specific breed distributions may influence the pathogenesis of this disease. Furthermore, the lists of endocrine cancer predisposed breeds were not UK specific, but included data from studies that were conducted outside the UK. Additionally, these endocrine cancer predisposed breed lists may have lacked specificity due to reliance on prior studies that used different statistical methods with varying statistical power. Still, the reported association provides a rationale to design future prospective multi-institutional studies investigating the etiopathogenesis of CEN in dogs. These studies should focus on investigating the presence of mutations in the *MEN1*, *NF-1*, and *TSC1/TSC2* genes, because insulinomas can develop in the context of the genetic tumor syndromes that occur due to mutations in these genes^47^.

In addition to the limitations discussed above, the current study had some other limitations associated witfh the secondary use of veterinary EHRs as a research resource. These limitations included issues such as missing data and variations in the rigor and quality of the available clinical records. Some of the dogs determined to have insulinomas by a primary care veterinarian may have been misclassified and thus posed a limitation to the study due to possible false positive diagnosis. Additionally, given that only around 30% of UK veterinary practices are collaborating with VetCompass, the current results may not generalize fully to all UK practices, although the UK veterinary practices collaborating with VetCompass include practices of varying sizes, geographical locations and caseloads^48^. Unmeasured confounding factors such as socio-economic status of owners, regional differences in veterinary care and environmental factors, and differences in physical activity levels of dogs may also have influenced the results of the current study. Finally, this study did not apply corrections for multiple testing in the various analyses carried out, as this was an exploratory study in nature that can support future hypothesis generation but where the current study did not involve testing a predefined hypothesis. It should be noted that failure to correct for multiple testing increases the probability of finding at least one result to be statistically significant just by chance (the problem of multiplicity), which should be considered when interpreting the results^49^.

In conclusion, this is the first epidemiological study on canine insulinoma in the UK dog population under primary veterinary care and reported an annual incidence risk of 0.003% and an annual prevalence of 0.004%. Nine risk factors were associated with canine insulinoma: breed, sex/neuter, age, median adult bodyweight, median adult bodyweight compared to the median for sex/breed, terrier breed, breed predisposed for parathyroid cancer, breed predisposed for thyroid cancer and breed predisposed for parathyroid and/or thyroid cancer. Dogue de Bordeaux, German Pointer, Flat Coated Retriever, Boxer, Hungarian Vizsla and West Highland White Terrier were identified as breeds with ultra-predisposition for insulinoma diagnosis compared with crossbreeds. These findings of risk factors and breed predispositions can contribute to improved recognition of cases of canine insulinoma and enhance future understanding of insulinoma tumorigenesis.

## Methods

The study population included 2,250,741 dogs under primary veterinary care in 2019 at practices across the UK participating within the VetCompass program^28^. VetCompass collates de-identified EHR data from veterinary practices in the UK for epidemiological research^28^. Dogs under veterinary care were defined as those with ≥ 1 EHR (free-text clinical note, treatment, or bodyweight) recorded in 2019. Data fields available to VetCompass researchers include a unique animal identifier along with species, breed, date of birth, sex and neuter status and also clinical information from free-form text clinical notes, bodyweight and treatment with relevant dates^28^. Of the dogs with ≥ 1 EHR recorded in 2019, cohort EHR data recorded up to August 31, 2023 were accessible for the current study.

A retrospective cohort study design was used to estimate the annual (2019) prevalence and annual (2019) incidence risk for diagnosis with insulinoma and to identify associations between demographic risk factors and insulinoma diagnosis at any date in the available clinical records. Based on a preliminary screen suggesting a crude prevalence for insulinoma of 0.01% within VetCompass, power calculation estimated that at least 150,747 dogs were needed to estimate the frequency for a disorder occurring in 0.01% of dogs with 0.005% acceptable margin of error at a 95% confidence level from an estimated national UK population of 8 million dogs^50,51^. Ethical approval was granted by the RVC Social Science Research Ethical Review Board (SSRERB) (reference number SR2018-1652).

Insulinoma cases were defined as having at least one of the following inclusion criteria:

1. Evidence of a recorded final diagnosis of insulinoma in available EHRs.
2. Histopathological confirmation of insulinoma reported in available EHRs.
3. Concurrent occurrence of blood glucose < 4.2 mmol/L, plasma insulin > 10 µU/mL and matching clinical signs of hypoglycemia in available EHRs.
4. Evidence of concurrent hypoglycemia, a pancreatic mass lesion on diagnostic imaging, matching clinical signs of hypoglycemia and absence of systemic inflammatory response syndrome in available EHRs.

Case-finding involved two phases. Firstly, candidate cases for insulinoma were identified by searching the clinical notes using the search terms: ‘insulinoma*’, ‘*diazoxide*’, ‘*blood insulin*’, ‘*serum insulin*’, ‘*octreotide*’, ‘*streptozocin*’ and ‘*streptozotocin*’ and the treatment fields using the search terms: ‘*diazoxide*’ and ‘*eudemine*’. The clinical notes of all candidate cases were manually reviewed to evaluate for case inclusion. Non-cases of insulinoma used in the risk factor analysis included a random sample of 750,000 dogs not identified as candidate cases.

Following data cleaning in Excel (Microsoft Office Excel 2016), statistical analysis used SPSS^®^ Statistics version 29 (IBM^®^). Breed descriptive information entered by the participating practices was cleaned and mapped to a VetCompass breed list derived and extended from the VeNom Coding breed list that included both recognized purebred breeds and also designer breed terms^52^. A breed purity (named *Purebred*) variable categorized all dogs of recognizable breeds as ‘purebred’, dogs with contrived names generated from two or more purebred breed terms as ‘designer’ crossbreds (purposely bred crossbreeds) and dogs recorded as mixes of breeds but without a contrived name as ‘crossbred’^53^. A *Breed* variable retained individual pure breeds and designer hybrids represented by ≥ 2 insulinoma cases, along with a grouping of all remaining breeds and also a grouping of general crossbred dogs. This approach was taken to facilitate statistical power for the individual breed analyses^54^. A *terrier* variable distinguished terrier breeds assigned to the breed group ‘terrier’ and other breeds as ‘non-terrier’ following a combined classification according to the Kennel Club and VeNom Coding Group, as shown in Supplementary Table 1^52,53^. A list of breeds predisposed for various types of endocrine cancer was created within the current study based on evidence sourced from multiple studies. Studies were selected based on having a reasonable study population size and, preferably, using validated statistical models, implicating sufficient statistical power (Supplementary Table 2). Based on this list, multiple variables were created, including *Breed predisposed for parathyroid cancer*, *Breed predisposed for thyroid cancer*, *Breed predisposed for parathyroid- and/or thyroid cancer*, *Breed predisposed for pituitary/adrenal gland cancer*, *Breed predisposed for parathyroid- and/or thyroid- and/or pituitary/adrenal gland cancer*, *Breed predisposed for mammary gland cancer*, *Breed predisposed for testicular/ovarian cancer*, *Breed predisposed for reproductive endocrine cancer* and *Breed predisposed for one or multiple types of endocrine cancer*.

Sex and neuter status were defined by the final available EHR value and were combined into a single variable for the risk factor analysis. Median adult bodyweight was defined as the median of all bodyweights (kg) values recorded for each dog after reaching 18 months of age and was categorized as: <10.0, 10.0 to < 20.0, 20.0 to < 30.0 and ≥ 30.0. Age at first diagnosis was generated from the date of first insulinoma diagnosis and date of birth. Age of the non-cases was defined at December 31, 2019. Age was categorized in six categories for multivariable analysis: less than six years, six to less than nine years, nine to less than twelve years, twelve to less than fifteen years, fifteen years and older and unrecorded. Continuous variables that were normally distributed were summarized using mean (standard deviation [SD]) and for non-normally distributed data as median (interquartile range [IQR] and range).

Lifetime prevalence for insulinoma diagnosis was calculated as the total number of cases at any date in all available EHRs up to August 31, 2023 divided by the total number of dogs in the study. The one-year (2019) period prevalence was estimated by dividing all dogs that met the case definition at any date up to December 31, 2019 by the denominator population described above. The annual incidence risk was calculated as the number of cases newly diagnosed in 2019 divided by the total number of dogs. Breed lifetime prevalence was estimated similarly, by dividing the number of cases of a breed within the lifetime insulinoma group by the number of dogs of that breed within the denominator population. The confidence intervals (CI) for the prevalence and incidence values were derived from standard errors, based on approximation to the binomial distribution. Risk factor analysis included all cases diagnosed at any date to August 31, 2023 (i.e. available lifetime risk) to maximize statistical power. Binary logistic regression modelling was used to evaluate univariable associations between risk factors for diagnosis (sex/neuter, breed, purebred status, median adult bodyweight, age, median adult bodyweight in relation to the median for the sex/breed, being a terrier breed, being a breed predisposed for parathyroid cancer, being a breed predisposed for thyroid cancer, being a breed predisposed for parathyroid- and/or thyroid cancer, being a breed predisposed for pituitary/adrenal gland cancer, being a breed predisposed for parathyroid- and/or thyroid- and/or pituitary/adrenal gland cancer, being a breed predisposed for mammary gland cancer, being a breed predisposed for testicular/ovarian cancer, being a breed predisposed for reproductive endocrine cancer and being a breed predisposed for one or multiple types of endocrine cancer) and an outcome of being a dog under primary veterinary care in 2019 and having a diagnosis of insulinoma at any date to August 31, 2023. Risk factors with liberal association (*P*-value < 0.2) in univariable modelling were taken forwards for consideration in multivariable modelling^55^. Multivariable logistic regression modelling used automated SPSS model-building. Median adult bodyweight is a defining characteristic of individual breeds and therefore was excluded from consideration in initial multivariable breed-focused modelling^56^. Other variables derived directly from the breed variable (*Purebred*,* Terrier*,* Breed predisposed for parathyroid cancer*, *Breed predisposed for thyroid cancer*, *Breed predisposed for parathyroid- and/or thyroid cancer*, *Breed predisposed for pituitary/adrenal gland cancer*, *Breed predisposed for parathyroid- and/or thyroid- and/or pituitary/adrenal gland cancer*, *Breed predisposed for mammary gland cancer*, *Breed predisposed for testicular/ovarian cancer*, *Breed predisposed for reproductive endocrine cancer* and *Breed predisposed for one or multiple types of endocrine cancer)* were similarly not considered in initial breed-focused multivariable modelling. Instead, these variables individually replaced the *Breed* variable in the final breed-focused multivariable model to evaluate their effects after taking account of the other variables in that model^57^. The area under the ROC curve was used to evaluate discrimination of the final breed-focused model. Retention in the final multivariable logistic regression model and for statistical significance was set at *P* < 0.05.

## Electronic supplementary material

Below is the link to the electronic supplementary material.

Supplementary Material 1

## Data Availability

The datasets generated during the current study are available at Figshare: dx.doi.org/10.6084/m9.figshare.6025748.
