# Supplementary material for: Incidence and risk factors for insulinoma diagnosed in dogs under primary veterinary care in the UK
Source: Sci Rep. 2025 Jan 20;15:2463. doi: 10.1038/s41598-025-86782-6 (PMC11743789; doi:10.1038/s41598-025-86782-6)
Supplement: Supplementary file 1 — Supplementary Material 1 [file 41598_2025_86782_MOESM1_ESM.docx]

**Supplementary Information**

*Supplementary Table T1* – Breeds classified as terrier breed. Breeds not included below are classified as non-terrier.

| **Breed** | | |
| --- | --- | --- |
| Aberdeen Terrier | Fell Terrier | Russian Toy Terrier |
| Airedale Terrier | Fox Terrier | Scottish Terrier |
| American Bull Terrier | German Hunting Terrier | Sealyham Terrier |
| American Pit Bull Terrier | Glen of Imaal Terrier | Shropshire Terrier |
| Atlas Terrier | Irish Staffordshire Bull Terrier | Skye Terrier |
| Australian Silky Terrier | Irish Terrier | Smooth Fox Terrier |
| Australian Terrier | Jack Russell Terrier | Soft Coated Wheaten Terrier |
| Bedlington Terrier | Kerry Blue Terrier | Staffordshire Bull Terrier |
| Biewer Terrier | Lakeland Terrier | Standard Manchester Terrier |
| Black Fell Terrier | Lukas Terrier | Tenterfield Terrier |
| Black Russian Terrier | Manchester Terrier | Terrier (unspecified) |
| Border Terrier | Miniature Bull Terrier | Tibetan Terrier |
| Brazilian Terrier | Moscow Toy Terrier | Toy Manchester Terrier |
| Bull Terrier | Norfolk Terrier | Toy Terrier |
| Cairn Terrier | Norwich Terrier | Welsh Terrier |
| Cesky Terrier | Parson Russell Terrier | West Highland White Terrier |
| Cyprus Terrier | Patterdale Terrier | Wire-Haired Fox Terrier |
| Dandie Dinmont Terrier | Plummer Terrier | Wire-Haired Terrier |
| English Bull Terrier | Red Fell Terrier | Yorkshire Terrier |
| English Toy Terrier | Russell Terrier |  |

### *Supplementary Table 2–*- Breeds predisposed for certain types of endocrine cancer.

The following supplementary table includes the results of literature research identifying breed predispositions for types of endocrine cancer, with comments added below. The reference list is displayed in Supplementary References R1.

| Breed | Type of cancer | References according to Supplementary References R1 |
| --- | --- | --- |
| Dachshund | Mammary gland, parathyroid, testicular | [1-3] |
| English Setter | Mammary gland, parathyroid | [1, 2] |
| English Springer Spaniel | Mammary gland, parathyroid | [1, 2] |
| Lhasa Apso | Mammary gland, pituitary/adrenal glands, parathyroid | [1, 2, 4] |
| Boxer | Mammary gland, pituitary/adrenal glands, Thyroid, testicular | [1, 5-7] |
| Bichon Frise | Mammary gland, pituitary/adrenal glands | [1, 4, 8] |
| Jack Russell Terrier | Mammary gland, pituitary/adrenal glands | [1, 4, 5] |
| Yorkshire Terrier | Mammary gland, pituitary/adrenal glands | [1, 4, 5] |
| Staffordshire Bull Terrier | Mammary gland, pituitary/adrenal glands | [1, 4] |
| English Cocker Spaniel | Mammary gland, testicular | [1, 9] |
| German Shepherd | Mammary gland, testicular | [1, 7] |
| Labrador Retriever | Mammary gland | [1] |
| German Shorthaired Pointer | Mammary gland | [1] |
| Greyhound | Mammary gland | [1] |
| Doberman Pinscher | Mammary gland | [1] |
| Pomeranian | Mammary gland | [1] |
| Lurcher | Mammary gland | [1] |
| Pointer | Ovarian | [10] |
| English Bulldog | Ovarian | [10] |
| Norwegian Elkhound | Parathyroid, Testicular | [2, 3] |
| Keeshond | Parathyroid | [2] |
| Rhodesian Ridgeback | Parathyroid | [2] |
| Australian Shepherd | Parathyroid | [2] |
| Briard | Parathyroid | [2] |
| Irish Setter | Parathyroid | [2] |
| Wirehaired Fox Terrier | Parathyroid | [2] |
| American Eskimo | Parathyroid | [2] |
| Shih Tzu | Pituitary/adrenal glands, parathyroid | [2, 5] |
| Standard Schnauzer | Pituitary/adrenal glands | [5] |
| Fox Terrier | Pituitary/adrenal glands | [5] |
| Cavalier King Charles Spaniel | Pituitary/adrenal glands | [5] |
| Pit Bull | Pituitary/adrenal glands | [5] |
| Bolognese | Pituitary/adrenal glands | [5] |
| Maltese | Pituitary/adrenal glands | [5] |
| Miniature Dachshund | Pituitary/adrenal glands | [5] |
| Miniature Poodle | Pituitary/adrenal glands | [5] |
| Border Terrier | Pituitary/adrenal glands | [4] |
| Miniature Schnauzer | Pituitary/adrenal glands | [4] |
| Poodle (incl. Miniature and standard) | Testicular | [3] |
| Golden Retriever | Thyroid, parathyroid, testicular | [2, 9, 11] |
| Siberian Husky | Thyroid, parathyroid, testicular | [2, 3, 11] |
| Shetland Sheepdog | Thyroid, Testicular | [3, 11] |
| Beagle | Thyroid | [11] |

Note: breeds predisposed for hypercortisolism are classified as pituitary/adrenal glands to include breeds that are at increased risk of developing hypercortisolism and therefore have an endocrine tumor (although not specified if originating from the pituitary gland or adrenal glands).

*Supplementary references R1*

1. Varney, D. *et al.* Epidemiology of mammary tumours in bitches under veterinary care in the UK in 2016. *Veterinary Record* **193**, (2023).
2. Refsal, K. R., Provencher-Bolliger, A. L., Graham, P., Cert, V. R. & Nachreiner, R. F. Update on the Diagnosis and Treatment of Disorders of Calcium Regulation. *Veterinary Clinics of North America: Small Animal Practice* **31**, 1043–1062 (2001).
3. Nødtvedt, A. *et al.* Breed differences in the proportional morbidity of testicular tumours and distribution of histopathologic types in a population‐based canine cancer registry. *Veterinary and Comparative Oncology* **9**, 45–54 (2010).
4. Schofield, I. *et al.* Frequency and risk factors for naturally occurring Cushing’s syndrome in dogs attending UK primary‐care practices. *Journal of Small Animal Practice* **63**, 265–274 (2021).
5. Carotenuto, G. *et al.* Cushing’s syndrome—an epidemiological study based on a canine population of 21,281 dogs. *Open Veterinary Journal* **9**, 27 (2019).
6. Hayes, H. M. & Fraumeni, J. F. Canine thyroid neoplasms: Epidemiologic Features 2. *JNCI: Journal of the National Cancer Institute* **55**, 931–934 (1975).
7. Grieco, V. *et al.* Canine Testicular Tumours: a Study on 232 Dogs. *Journal of Comparative Pathology* **138**, 86–89 (2008).
8. O’Neill, D. G. *et al.* Epidemiology of hyperadrenocorticism among 210,824 dogs attending primary‐care veterinary practices in the UK from 2009 to 2014. *Journal of Small Animal Practice* **57**, 365–373 (2016).
9. Manuali, E. *et al.* A five-year cohort study on testicular tumors from a population-based canine cancer registry in central Italy (Umbria). *Preventive Veterinary Medicine* **185**, 105201 (2020).
10. Hayes, H. M. & Young, J. L. Epidemiologic features of canine ovarian neoplasms. *Gynecologic Oncology* **6**, 348–353 (1978).
11. Wucherer, K. L. & Wilke, V. L. Thyroid cancer in dogs: An update based on 638 cases (1995–2005). *Journal of the American Animal Hospital Association* **46**, 249–254 (2010).
